# How eggs arrest at metaphase II: MPF stabilisation plus APC/C inhibition equals Cytostatic Factor

**DOI:** 10.1186/1747-1028-2-4

**Published:** 2007-01-26

**Authors:** Suzanne Madgwick, Keith T Jones

**Affiliations:** 1 Institute for Cell and Molecular Biosciences, The Medical School, University of Newcastle, Newcastle NE2 4HH, England, UK

## Abstract

Oocytes from higher chordates, including man and nearly all mammals, arrest at metaphase of the second meiotic division before fertilization. This arrest is due to an activity that has been termed 'Cytostatic Factor'. Cytostatic Factor maintains arrest through preventing loss in Maturation-Promoting Factor (MPF; CDK1/cyclin B). Physiologically, Cytostatic Factor – induced metaphase arrest is only broken by a Ca^2+ ^rise initiated by the fertilizing sperm and results in degradation of cyclin B, the regulatory subunit of MPF through the Anaphase-Promoting Complex/Cyclosome (APC/C). Arrest at metaphase II may therefore be viewed as being maintained by inhibition of the APC/C, and Cytostatic Factor as being one or more pathways, one of which inhibits the APC/C, consorting in the preservation of MPF activity.

Many studies over several years have implicated the c-Mos/MEK/MAPK pathway in the metaphase arrest of the two most widely studied vertebrates, frog and mouse. Murine downstream components of this cascade are not known but in frog involve members of the spindle assembly checkpoint, which act to inhibit the APC/C. Interesting these downstream components appear not to be involved in the arrest of mouse eggs, suggesting a lack of conservation with respect to c-Mos targets. However, the recent discovery of Emi2 as an egg specific APC/C inhibitor whose degradation is Ca^2+ ^dependent has greatly increased our understanding of MetII arrest. Emi2 is involved in both the establishment and maintenance of metaphase II arrest in frog and mouse suggesting a conservation of metaphase II arrest. Its identity as the physiologically relevant APC/C inhibitor involved in Cytostatic Factor arrest prompted us to re-evaluate the role of the c-Mos pathway in metaphase II arrest.

This review presents a model of Cytostatic Factor arrest, which is primarily induced by Emi2 mediated APC/C inhibition but which also requires the c-Mos pathway to set MPF levels within physiological limits, not too high to induce an arrest that cannot be broken, or too low to induce parthenogenesis.

## Background

**Figure 1:**
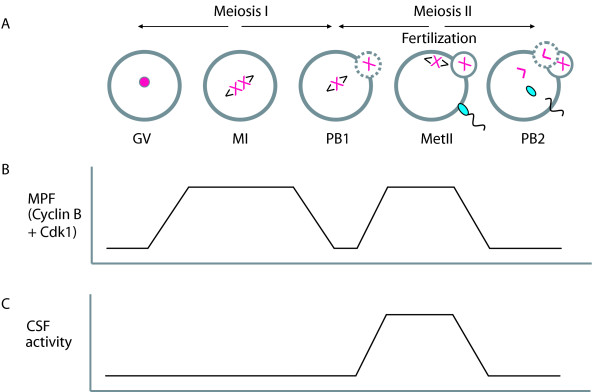
**The events of female meiosis**. (A) Only one pair of homologous chromosomes is shown. After S-phase two cell divisions are required to produce a haploid gamete. During MI, homologous chromosomes segregate between the egg and the first polar body. On MI completion, eggs arrest their cell cycle at MetII. MetII exit is blocked through CSF activity, until sperm break the arrest. Eggs complete MII and in so doing segregate sister chromatids and extrude a second polar body. (B) MPF activity oscillates in time with entry to, and exit from metaphase. (C) At MetII eggs arrest their cell cycle with high levels of CSF activity.

Meiosis is a process in which two consecutive cell divisions (MI and MII) occur in the absence of an intervening S-phase. MI is a reductional division in which homologous chromosomes are segregated, sister chromatids are only resolved following the equational MII division (Fig 1a). On completion of MI, oocytes prevent parthenogenetic activation by arresting their cell cycle at metaphase of MII (MetII) due to an activity termed Cytostatic Factor (CSF) [1,2]. CSF blocks MetII exit until sperm break arrest via a cytoplasmic Ca^2+ ^signal [3-5] which induces completion of MII.

Maturation (or M-Phase) Promoting Factor (MPF; CDK1/cyclin B) [6,7] activity drives somatic cells into mitosis and eggs into meiosis (for reviews see [8-10]). MPF is regulated during meiosis, oscillating in time with entry to, and exit from MI and MII (Fig 1b). The activity of MPF can be regulated by both CDK1 phosphorylation and cyclin B degradation (for reviews see [2,11]). Hereafter cyclin B is used to denote any B-type cyclin degraded at metaphase, in frog this constitutes B1, B2, B4 and B5, while in mammals B1 and B2 [12,13]. Most is known about cyclin B1 and B2 in frog and cyclin B1 in mammals. Mammalian cyclin B1 appears to be particularly important for eggs in Met II arrest, whereas B2 is non-essential and in all of this review, the use of cyclin B in the context of mammalian eggs actually refers to work carried out using cyclin B1 [8,14,15]. At M-phase exit, as would occur at fertilization, loss of MPF is normally associated with the rapid destruction of cyclin B by anaphase [16] since CDK1 has no catalytic function without its regulatory partner [17]. Cyclin B is degraded by a Destruction-box motif (D-box) in its primary sequence, which is recognized by the E3 ligase Anaphase Promoting Complex/Cyclosome (APC/C). The APC/C polyubiquitinates key cell cycle proteins such as cyclin B, targeting them for immediate proteolysis by the 26S proteasome [18-20].

Eggs arrest at MetII with high MPF due to CSF activity (Fig 1c). The long-term stability of MPF is unique to eggs since in a mitotic metaphase, the APC/C would be active and cyclin B degraded. Although it is possible to exit CSF mediated MetII arrest by inhibiting the CDK1 component of MPF [21,22], physiologically a sperm Ca^2+ ^signal induces loss of cyclin B rather than CDK1 inactivation. Interestingly in mouse eggs, the APC/C is not completely inhibited during MetII arrest, such that eggs rely on continual cyclin B synthesis to maintain arrest [3,23,24]. Similarly in frog eggs, the APC/C remains active enough to degrade cyclin B [25]. At fertilization, Ca^2+ ^stimulates APC/C activity, in mouse about 6-fold [23], such that cyclin B degradation results in MPF loss. Blocking cyclin B degradation by D-box mutation prevents exit from MetII despite a Ca^2+ ^signal [26,27].

Unlike MPF, the identity of CSF has never been fully resolved, despite simultaneous identification of both activities in a seminal paper [28]. Observations regarding the relationship between MPF and APC/C activity have led to the conclusion that CSF activity is likely to constitute an APC/C inhibitor [2]. In mitosis, most is known about how the APC/C is inhibited by the spindle assembly checkpoint (SAC) proteins therefore we first discuss evidence that CSF activity is due to activation of the SAC pathway.

## SAC proteins as CSF

SAC proteins were identified in budding yeast mutants that lost ability to metaphase arrest after addition of spindle poisons [29,30]. SAC proteins function in arresting cells in metaphase by inhibiting the APC/C until all chromosomes are biorientated and so under tension from spindle microtubules (reviews see [31-33]). Vertebrate homologues of the SAC proteins Bub1 (Budding uninhibited by benzimidazole 1) Mad1 and Mad2 (Metaphase arrest deficient 1 and 2) have been suggested to affect MetII arrest in *Xenopus *eggs. Immunodepletion of these SAC proteins from egg extracts have all been demonstrated to block CSF arrest [34,35].

SAC components have been implicated as the downstream effectors the c-Mos/MEK/MAPK/p90rsk pathway, long thought to be essential for frog CSF arrest. c-Mos (pp39mos), a proto-oncogene from a family of kinases functioning in signal transduction regulating cell growth and differentiation [36], is highly expressed during germ cell maturation, and has proposed roles throughout frog oocyte maturation [37-43]. Functioning as a MAPK kinase kinase (MEKK), c-Mos is important for activation of the MAPK kinase, MEK1 [44-46]. MEK1 serves as the upstream activator of MAPK [47-49], which switches on the 90-kD ribosomal protein S6 kinase (p90rsk [50]). At fertilization c-Mos is degraded [43], whilst MEK1, MAPK and p90rsk are inactivated shortly afterwards [51,52].

The c-Mos ...p90rsk signaling cascade has been shown to aid directly MPF activation and stabilization [53-55] making it an ideal CSF candidate. Microinjection of c-Mos RNA into two-cell embryos results in metaphase arrest, and immunodepletion of c-Mos causes a loss of cleavage-arresting activity [43]. Similarly, an injection of an active form of MAPK [56] or constitutively-active rsk [57], into blastomeres of two-cell embryos arrests the injected blastomere in metaphase. Indeed in frog, p90rsk has been suggested to be the only MAPK substrate needed for cyclin B re-accumulation on entry to MetII, MetII spindle formation, and CSF arrest [57,58]. This is supported by the fact that c-Mos protein is unable to establish CSF arrest in frog egg extracts immunodepleted of p90rsk [59].

The SAC component Bub1 is phosphorlyated and activated by p90rsk [60]. Bub1 [34], Mad1 and Mad2 [35] all appear to be required downstream of c-Mos given that the immunodepletion of these proteins blocked the establishment of CSF arrest by c-Mos in frog egg extracts. Such studies suggest a model in which CSF arrest by c-Mos is mediated by Bub1, Mad1 and Mad2 proteins.

From the above it appears that a well-defined CSF pathway has been identified in frog. However, studying SAC components maybe somewhat misleading with respect to identifying CSF. Although CSF activity and the SAC are similar in being able to induce metaphase arrest through APC/C inhibition, they may use different signalling pathways. Any arrest must be reversed by Ca^2+ ^to prove physiological relevance with respect to CSF. A further issue is how CSF arrest can be achieved at MetII but not at MI metaphase (MetI) since many components of the c-Mos pathway are present and active at MetI. For example, c-Mos, MAPK, p90rsk and Bub1 are essential for suppression of S-phase between meiotic divisions [43,58,61,62] yet do not block eggs at MetI. A possible explanation is the involvement of cyclin E/cdk2, both of which are synthesized during MII [63] and inhibit the APC/C.

In frog eggs cyclin E/Cdk2 activity has been reported to play an essential role in CSF arrest. Cyclin E/Cdk2, like c-Mos, can establish metaphase arrest in egg extracts [34,63]. Cdk2 antisense prevents CSF arrest [64] and recombinant cyclin E/Cdk2 causes metaphase arrest in egg extracts even in the absence of c-Mos [34]. The two pathways (c-Mos and cyclin E/cdk2) are therefore suggested to be independent of each other but both appear to inhibit the APC/C. CSF activity therefore may result from the coexpression of cyclin E/Cdk2 with the c-Mos/MEK/MAPK/p90rsk pathway. However, the role of cyclin E/cdk2 in CSF arrest remains to be fully elucidated since inhibiting cdk2 [65], and ablation of cyclin E [66] have both been reported not disrupt CSF arrest.

Once CSF arrest has been established then many of the above proteins seem no longer required for maintenance (p90rsk, Mad2, Bub1 and cyclin E/cdk2 are all dispensable for maintenance [34,35,59]). This suggests that these proteins act upstream or independently of other effectors of CSF activity. SAC proteins may be essential to improve the efficiency of APC/C inhibition on entry into MetII arrest, yet appear redundant in the maintenance of arrest.

Whilst the c-Mos/MEK/MAPK/p90rsk/(SAC proteins) pathway is well established in the frog, its role in mammalian eggs is less clear. Although eggs from c-Mos knockout mice eventually undergo parthenogenetic activation [67,68], they do MetII arrest, remaining there for 2–4 h, before going on to exit MII [69]. This suggests that whilst c-Mos is critical for protracted MetII arrest, it is not required for its establishment. Loss of MEK or MAPK activity also results in parthenogenesis [21] suggesting as in frog they are downstream components of the c-Mos pathway. However, p90rsk plays no essential role in mouse because eggs from Rsk knockouts arrest at MetII [70]. Furthermore SAC proteins do not mediate CSF activity since mouse eggs expressing dominant negative mutants of Bub1 and Mad2 arrest at MetII [71]. Therefore the c-Mos/MEK/MAPK pathway acting independently of p90rsk is likely only to be involved in helping maintaining MetII arrest in mammals, rather than having a direct role in its establishment.

## Emi2 as CSF

When considering all of the above, one may conclude that the c-Mos pathway is unlikely to constitute fully CSF. Recently an egg-specific protein Emi2 (or Early mitotic inhibitor 1-related protein 1; Erp1) has been identified. Emi2 degradation is Ca^2+ ^dependent and likely functions to both establish and maintain CSF arrest by APC/C inhibition [72-76]. Interest in Emi2 was generated from work on a related protein Emi1, which prevents premature APC/C activation in G2 of the mitotic cell cycle by binding to the APC/C activator protein Cdc20 [77]. Although Emi1 itself was initially suggested to be involved in MetII arrest [78], this is now known not to be so [79], and was probably due to antibody cross-reactivity between the two Emi proteins [76].

Emi2 is a substrate of a polo-like kinase (Plk), which plays a crucial role in regulating progression through M phase [80], allowing timely activation of the APC/C at the onset of anaphase [81-83]. In frog eggs a role for Plx1 in Ca^2+^-mediated APC/C activation was demonstrated several years ago [81] with the authors proposing the existence of a Plx1-regulated inhibitor of the APC/C active at MetII which was inactivated by Plx1 at fertilization. A later yeast-two hybrid screen for Plx1-interacting proteins identified Emi2. Like CSF activity, Emi2 accumulates during egg maturation, is present and stable in CSF arrested egg extracts, but is rapidly degraded on Ca^2+ ^addition [75]. Plx1 is fully active at metaphase [84], yet at MetII it does not remove inhibition of the APC/C until at fertilization. At fertilization the target of Ca^2+ ^is calmodulin-dependent protein kinase II (CamKII) [85,86], which acts a priming kinase, directly phosphorylating Emi2 [72]. Plx1 further phosphorylates Emi2 [72-74], to generate a degron which is recognised by the SCF ubiquitin ligase, resulting in Emi2 polyubiquitination and destruction [74]. Therefore APC/C inhibition is only removed once both CamKII and Plx1 are active.

In mammals, like frog, a Ca^2+ ^signal breaks MetII arrest through a signalling pathway involving CamKII and activation the APC/C [87-89]. Although the full mechanism of Emi2 degradation has not yet been demonstrated in mouse, like frog, mouse Emi2 contains specific motifs for phosphorylation by both Plk and CamKII. Given that ablation of Emi2 in MetII arrested mouse eggs results in parthenogenetic activation [90], it would appear that the target of CamKII in mouse eggs is also Emi2. Supporting a role in maintaining CSF activity, Emi2 is extremely stable in MetII eggs, yet rapidly degraded by Ca^2+ ^[91]. As predicted for an APC/C inhibitor, it follows that on release from MetII, Emi2 destruction precedes that of cyclin B [91]. These findings taken together demonstrate an essential role for Emi2 in the maintenance of MetII arrest.

On entry into MetII Emi2 is also important for APC/C inhibition, allowing cyclin B, and so MPF, accumulation. Emi2 levels are low in oocyte maturation, presumably to allow the APC/C to be active and permit passage through MI [91]. Emi2 morpholinos added to maturing mouse oocytes prevent cyclin B re-accumulation on entry into MII and eggs consequentially fail to form MetII spindles, eventually decondensing their chromatin[91]. In this work, MetII arrest was rescued by re-addition of Emi2, expression of a D-box mutant of cyclin B or by addition of nocodazaole to induce a SAC mediated arrest. Emi2 also appears to be essential for the establishment of arrest in frog eggs [92,93] suggesting a conserved mechanism in vertebrates.

## Conclusion: Our model, MPF stabilisation plus APC/C inhibition equals CSF

A unifying hypothesis would be useful which invokes most of the CSF candidates described in both mouse and frog (the c-Mos/MEK/MAPK/p90rsk/Bub1; Mad1 and Mad2; cyclin E1/cdk2 and Emi2). Having a completely unified mechanism however looks unlikely given that the Mos/MAPK pathway in mouse does not involve p90rsk. To establish whether Emi2 and the c-Mos pathway function independently remains important but here we go on to suggest a working model of how these two pathways interact.

**Figure 2:**
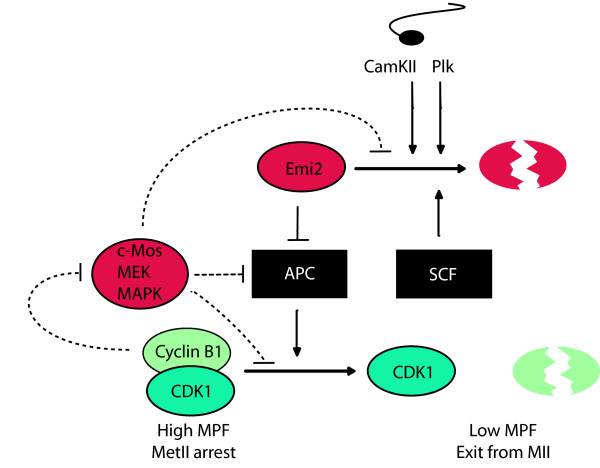
**Model of the regulation of MetII arrest in mammalian eggs**. High MPF activity is essential for MetII arrest and may be maintained via separate pathways; direct inhibition of the APC/C, and direct stabilization of MPF. The pathway which involves Emi2-mediated CSF arrest is shown in solid lines. In mouse eggs, the c-Mos pathway is not mediated by p90rsk, so its downstream targets remain obscure (dashed lines), but potential target points are shown as either inhibition of the APC/C or inhibition of Emi2 degradation. MPF activity may negatively regulate the c-Mos pathway, as based on studies from frog [94]. See text for further details.

We propose that MetII arrest is established through Emi2-mediated APC/C inhibition, and maintained both by Emi2 and the c-Mos/MAPK pathway, which acts to stabilise MPF (Fig 2). The exact nature of the effect of the c-Mos pathway on MPF stability is still to be fully resolved however Yamamoto et al. [94] in frog eggs showed that when MPF activity reaches a critical lower level, the c-Mos/MAPK pathway suppresses cyclin B degradation in order to elevate MPF levels; whilst elevation of MPF beyond a critical upper level activates APC/C dependent cyclin B degradation [94]. This suggests that Mos may help set the level of MPF activity. A CSF-arrested frog egg extract will exit MetII without cyclin B loss or a Ca^2+ ^stimulus when Greatwall kinase, known to positively affect MPF activity, is immunodepleted [95]. This illustrates the point that when considering the protacted nature of MetII arrest, one must consider mechanisms in the egg which are designed to respond to the sperm (Emi2 mediated APC/C inactivity) as well as those designed to keep MPF active until the time of fertilization. Our suggestion is that the c-Mos pathway may contribute to this second mechanism.

## List of abbreviations

APC/C Anaphase-Promoting Complex/Cyclosome

Bub, Budding uninhibited by benzimidazole

CamKII, Calmodulin-dependent protein kinase II

CSF, Cytostatic Factor

D-box, Destruction-box

Emi2, Early mitotic inhibitor 2

Mad, mitotic-arrest deficient

MI, first meiotic division

MII, second meiotic division

MetI, metaphase I

MetII, metaphase II

MPF, Maturation (M-Phase)-Promoting Factor; CDK1/cyclin B

p90rsk, 90-kD ribosomal protein S6 kinase

## Competing interests

The author(s) declare that they have no competing interests.

## Authors' contributions

SM wrote the review. Both authors contributed to the drafting of the text.
